# Clay-induced DNA breaks as a path for genetic diversity, antibiotic resistance, and asbestos carcinogenesis

**DOI:** 10.1038/s41598-018-26958-5

**Published:** 2018-05-31

**Authors:** Enrique González-Tortuero, Jerónimo Rodríguez-Beltrán, Renate Radek, Jesús Blázquez, Alexandro Rodríguez-Rojas

**Affiliations:** 1 Department of Ecosystem Research, Leibniz-Institute of Freshwater Ecology and Inland Fisheries (IGB), Müggelseedamm 301, 12587 Berlin, Germany; 2 Berlin Centre for Genomics in Biodiversity Research (BeGenDiv), Königin-Luise-Straße 6-8, 14195 Berlin, Germany; 3 Department of Microbial Biotechnology, Spanish National Center for Biotechnology, Calle Darwin 3, 28049 Madrid, Spain; 4 Evolutionary Biology, Institut für Biologie, Freie Universität Berlin, Berlin, Germany; 5 Present Address: Institute for Genome Sciences, University of Maryland Baltimore School of Medicine, 670 West Baltimore Street, 21201 Baltimore, MD USA

## Abstract

Natural clays and synthetic nanofibres can have a severe impact on human health. After several decades of research, the molecular mechanism of how asbestos induces cancer is not well understood. Different fibres, including asbestos, can penetrate cell membranes and introduce foreign DNA in bacterial and eukaryotic cells. Incubating *Escherichia coli* under friction forces with sepiolite, a clayey material, or with asbestos, causes double-strand DNA breaks. Antibiotics and clays are used together in animal husbandry, the mutagenic effect of these fibres could be a pathway to antibiotic resistance due to the friction provided by peristalsis of the gut from farm animals in addition to horizontal gene transfer. Moreover, we raise the possibility that the same mechanism could generate bacteria diversity in natural scenarios, playing a role in the evolution of species. Finally, we provide a new model on how asbestos may promote mutagenesis and cancer based on the observed mechanical genotoxicity.

## Introduction

Clays such as sepiolite are used together with antibiotics as growth promoters in farming. This practice improves animal growth and product quality, and these additives are common in feed for broiler chickens and pigs^1,2^. Sepiolite is considered to be safe, stable and chemically inert hence also being used in tablet formulation for human medicine^3^. However, a recent study demonstrated that clays used as animal feed additives can increase the risk of horizontal gene transfer (HGT) among microbes, possibly resulting in increased antibiotic resistance^4,5^.

The transformation of bacteria by foreign DNA can be achieved when clay fibres are spread by friction or vibrations. This phenomenon is known as the Yoshida effect^6^ and relies on the ability of mineral nanofibres or nano-needles to adsorb DNA and to penetrate bacterial cells under sliding friction forces^7^. By its mechanical nature, the Yoshida effect can be used to transform diverse bacterial species^5,8,9^. Sepiolite and other clays fibres are not only capable of delivering DNA into the receptor bacteria, the abrasive action of clays also means that they can promote the release of DNA by disrupting the bacterial cell envelope^5^.

Before Yoshida began his experiments with bacteria, the ability of asbestos to transform eukaryotic cells was reported at the end of the eighties^10^. Indeed, fibrous clays and industrial nanofibres are considered genotoxic and carcinogenic, likely due to their ability to damage DNA^11^. Fibrous clays have been assayed in several experimental models including bacteria and cell cultures, but they display a poor correlation with mutagenicity or carcinogenesis found *in vivo*^11,12^. According to these observations, a significant concern arises from fibrous clays or industrial nanofibres which are responsible for severe human diseases such as asbestosis^13,14^. However, short and long periods of exposure to fibres in several different genotoxicity tests have failed to identify a molecular basis of DNA damage^14^. Thus, to date, the mechanisms underlying the genotoxicity and carcinogenicity of asbestos and other fibres remain obscure.

Clays may also have the potential to enhance antibiotic resistance in farming activities^4^. In natural scenarios, sediments and stones (gastroliths) are frequently swallowed by animals resulting in the unavoidable exposure of their microbiota to pebbles, sand, and clays. Soils and waters are a primary source of antimicrobials, either by natural microbial production or environmental antibiotic pollution, a major selective pressure that favours resistant strains^15,16^. Gut microbes can themselves also produce antibiotic compounds^17^.

In this study, we experimentally demonstrate the ability of fibrous clays such as sepiolite and asbestos to transform bacteria and to induce mutagenic DNA double-strand breaks (DSBs) when they are exposed to friction forces. We propose a molecular mechanism of action for asbestos, which was a strong inducer of DSBs in *Escherichia coli* in the presence of friction. Finally, the importance of this mechanism is discussed for the speciation processes of the microbiota of animals that use gastroliths.

## Results and Discussion

### Sepiolite increases mutant frequency in *E. coli*

**Figure 1:**
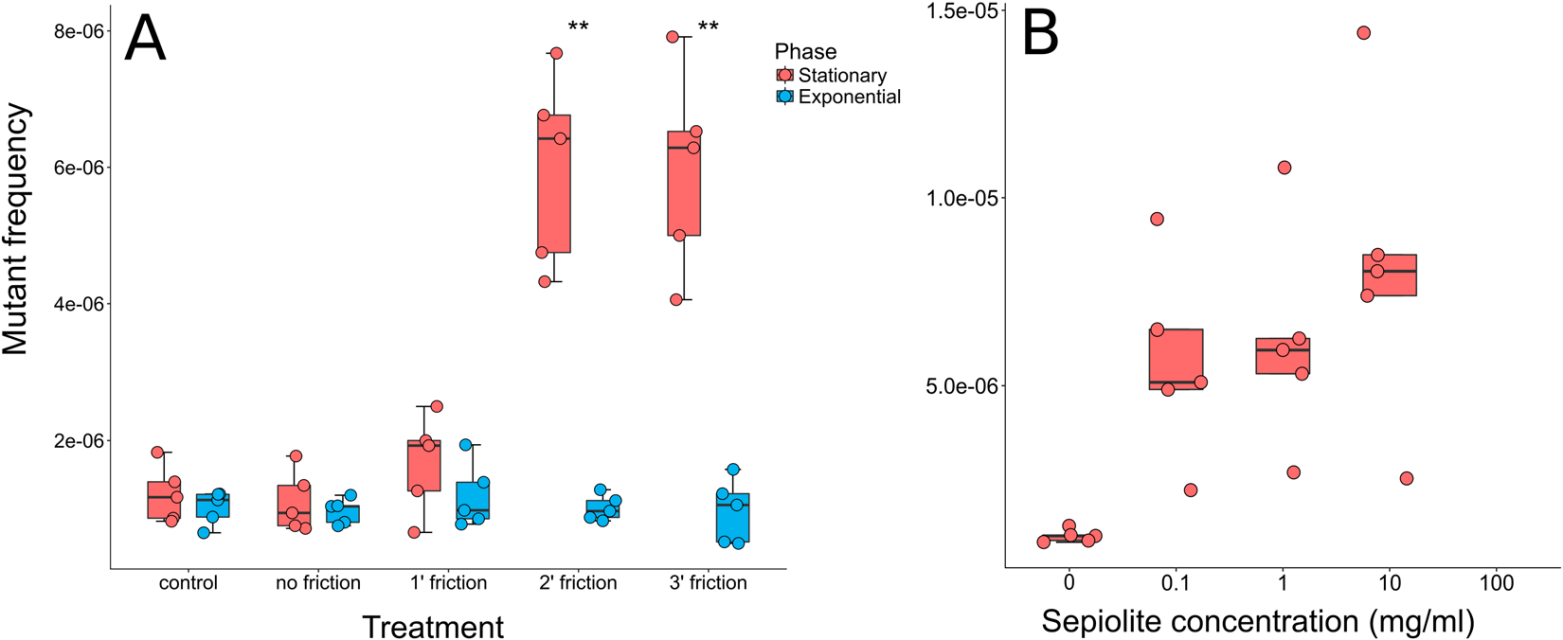
Sepiolite can induce mutagenesis after friction treatment only in stationary phase. Box-plot of the mutant frequency induced by sepiolite treatment in *E. coli* MG1655 exponential and stationary phases (**A**). The x-axis indicates the experimental treatment (control, the mixture of bacteria and sepiolite without friction force, and with friction force for one, two and three minutes). The increaase of sepiolite concentration increases the mutant frequency (**B**). There is a statistic significant donse effect response (P < 0.001, drc model fitted based on maximum likelihood). Asterisks in panel A represent significant differences; Mann-Whitney *U*: P < 0.01.

Different types of clays can transform bacteria by absorbing DNA and penetrating the cell envelope. In this case, the penetration could allow the clays to interact with the intracellular DNA and to promote mutations. To test whether the joint action of sepiolite and friction forces (as in transformation) has an impact on bacterial mutation rate, the mutant frequency of *Escherichia coli* was measured by plating the bacteria on agar containing the antibiotic fosfomycin, and enumerating spontaneous mutants. Different concentrations and friction times were tested (Fig. 1). When the cells were merely exposed to sepiolite without any friction on the agar plates’ surface, no significant differences in mutant frequencies were detected between them and control bacteria (Mann-Whitney U test; P = 0.999). In contrast, a six-fold increase in mutant frequency was found when friction was present for two or three minutes using 0.1 mg/ml of sepiolite (Mann-Whitney U test; P = 0.008) and a modest increase—but not significant—when the treatment lasted for one minute (Mann-Whitney U test; P = 0.421). Interestingly, only cells in the stationary phase displayed an increase in mutant frequency (Kruskal-Wallis test; P = 0.001): no significant mutagenesis was found when bacteria came from exponential cultures (Kruskal-Wallis test; P = 0.954; Fig. 1A). Regarding concentrations, we found the while sepiolite concentration increases, mutagenesis is also increased, and there is a statistic significant done-effect response (P = 2.2 × 10–16, DRC model fitted based on maximum likelihood, Fig. 1B). This result confirms that sepiolite is participating the mutagenicity of the treatments. We also noticed that although the trend in mutagenesis is highly significant, there are no significant differences among lower and high doses. The dispersion of data increased when more sepiolite was used. Also, the conservative inferences forced by the use of Bonferroni correction could influence the lack of detectable differences among lower and stronger doses. We chose the lower dose of 0.1 mg/ml for the rest of the experiments.

**Figure 2:**
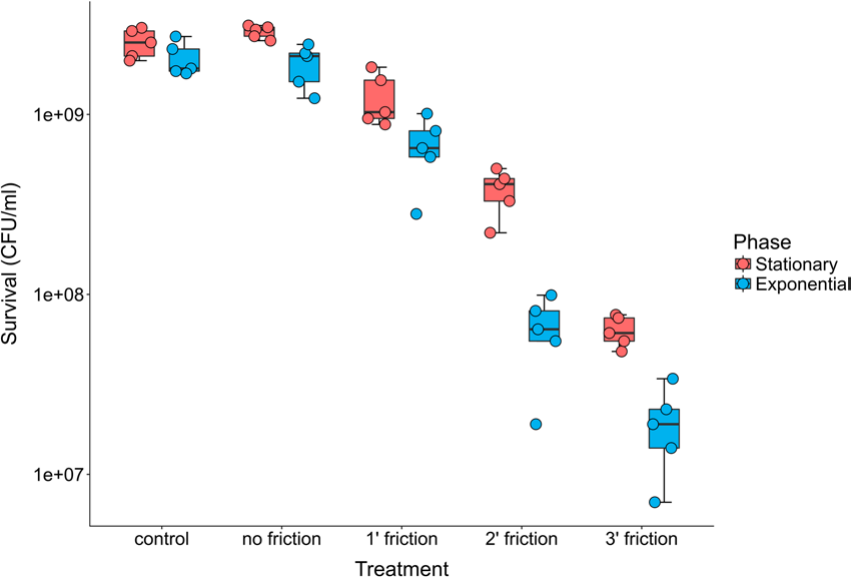
The longer the friction time, the lower the cell viability. Box-plot of the survival of *E. coli* MG1655 after the action of friction with sepiolite for one, two and three minutes of treatment. Groups with and without sepiolite gently spread with glass beads onto agar plates were used as controls.

Along with the mutant frequency experiments, the effect of the treatments on cell viability was checked. Sensitivity to the treatment was higher in the exponential phase than in the stationary phase cultures (Fig. 2). The observed mutant frequency differences were initially attributed to a higher sensitivity to the treatment. However, several hypotheses can explain this changes in mutant frequency and reduced-sensitivity in stationary phase cultures. The application of sliding force to bacterial cells with agar, in the absence of sepiolite or asbestos, did not account for any additional mutagenesis in the survival fraction (Fig. S1).

### Heavy metals have no influence on sepiolite mutagenesis

Many minerals containing metals such as iron, aluminium or copper are toxic to bacteria because of the generation of reactive oxygen species (ROS) via the Fenton reaction^18^. The release of metal ions inside the cell could, therefore, be the reason for the increase in mutagenesis. However, despite the addition of 2-2′ bipyridyl, a chelating agent, shortly before treatment, mutagenesis was still observed (Kruskal-Wallis test; P = 0.008; Fig. S2). This result indicates that the mutagenic effect does not depend on the metals present in the fibres.

### Sepiolite interacts with the DNA by causing double-strand breaks

**Figure 3:**
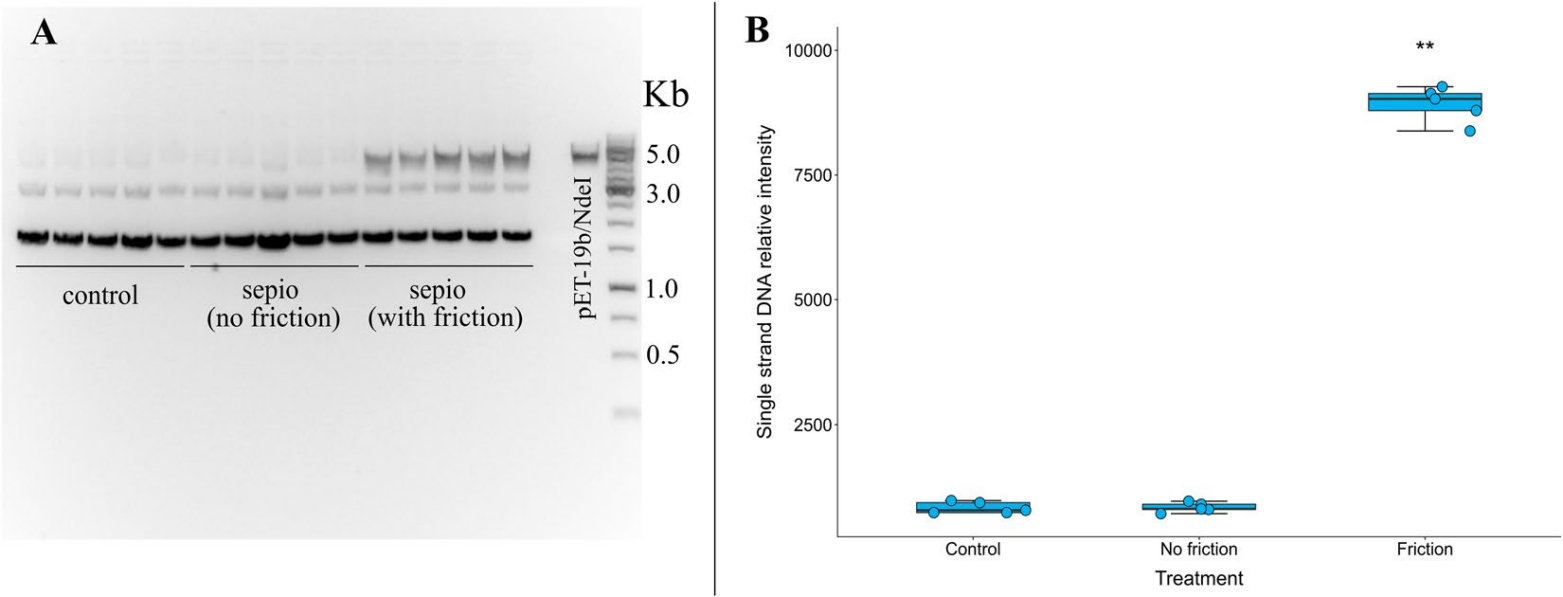
Linear plasmid DNA molecule abundance is higher when friction forces are applied. (**A**) Extraction of the plasmid pET-19b from sepiolite-treated *E. coli* DH5α, a *recA* deficient strain, after one minute of friction treatment (five extractions per treatment). Note the enrichment of linearised plasmid DNA molecules (5 Kb fragments?) from bacteria treated with sepiolite after two minutes of friction applied to a 1% agarose gel. (**B**) Box-plot of the abundance of single-strand DNA molecules after different experimental treatments (control, sepiolite without friction and sepiolite with friction). Plasmid pET-19b digested with a single cut site enzyme NdeI was used as a control for the linear molecule migration rate and as a reference to calculate relative intensities using a densitometry analysis. The asterisks represent a significant difference; Tukey HSD test: P < 0.01.

A second likely explanation for the mutagenesis could be the physical interaction between individual clay fibres in motion with DNA producing DSBs. The ability of sepiolite fibres to penetrate and interact with DNA has already been stated^6,19^. Physical or mechanical stress on the DNA duplex is a relevant cause of DSBs^20^. To evaluate this possibility, the *E. coli* DH5α strain (*recA* deficient) carrying the plasmid (pET-19b) was subjected to treatment with sepiolite and sliding friction. Sepiolite without friction and bacterial cells alone were used as controls. The plasmid content was extracted, and its integrity was evaluated by gel migration (Fig. 3A). DNA extracted from plasmids typically shows three molecular conformations: supercoiled DNA (which migrates quickly), nicked DNA (which is a closed circle but relaxed due to single strand breaks and it has an intermediate migration rate) and linear DNA molecules (with a lower migration speed)^21^. These latter DNA molecules were especially abundant in the friction-sepiolite treated group, whilst being present at a low level in control groups. Plasmids from the sepiolite group (under friction) had a significantly higher level of linearised molecules when compared to the control groups (One-Way ANOVA test; P = 3.33 × 10^−16^). According to these results, the joint action of sepiolite and friction are responsible for the induction of DSBs in the DNA. Interestingly, no increase in nicked DNA (single-strand break) was observed, indicating that if this type of lesion occurs, it is below the detection limit of this technique (Fig. 3B).

**Figure 4:**
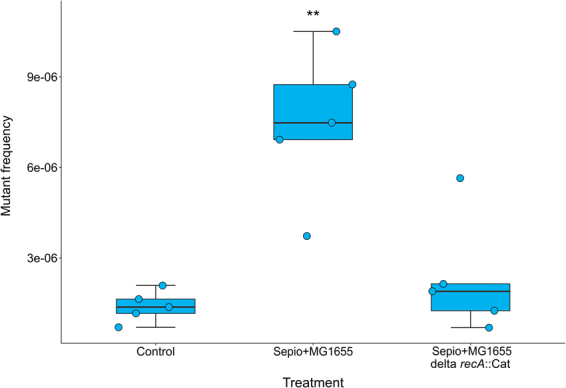
Inactivation of the *recA* gene suppresses the mutagenic effect of sepiolite under friction in *E. coli* MG1655. Box-plot of the mutant frequency of *E. coli* MG1655 and DH5α (derivative *recA* mutant) when treated with sepiolite for two minutes. The asterisks represent a significant difference; Mann-Whitney *U*: P < 0.01.

The view of mutagenic DSBs by mechanical shearing is consistent with the absence of mutagenic effect in exponentially growing bacteria. If the organism is diploid (even if the diploidy is only transient, as in replicating bacteria or replicating haploid yeast), then homology-directed repair can be used^20^. Because *E. coli* lacks a pathway to join non-homologous ends, homologous recombination is the only mechanism to salvage broken chromosomes^22^. But how can *E. coli* repair DSBs in stationary phase by homologous recombination? Stationary-phase cultures contain cells with several chromosome copies^23^. In exponentially growing *E. coli* DSB repair is non-mutagenic^24,25^. However, break repairs become mutagenic during the stationary phase and requires the Sigma S factor (RpoS), the SOS response, and the error-prone DNA polymerase PolIV. The change from one situation to the other has been described as a switch from high-fidelity repair in the exponential phase to error-prone DNA double-strand breaks during the stationary phase^24,25^. Because DSBs are lethal unless repaired, and repair action requires RecA protein^24,25^, the sepiolite mutagenesis experiment was repeated with *E. coli* DH5α, which is impaired in the SOS response triggering. We found that sepiolite mutagenesis was completely abolished by *recA* gene inactivation in the stationary phase (Kruskal-Wallis test; P = 0.011; Fig. 4). Thus, the lower level of mutant frequency in the *recA* deficient strain could be explained by the death of cells that suffered DSBs and were unable to repair them. Mutations introduced by DSB repair are considered a mechanism to generate diversity via mutagenic repair in bacteria^26,27^.

The mutagenicity of clay treatment is also potentially enhanced in stationary phase cells due to DNA being more tightly compacted than in the exponential phase^28^. Indeed, in *E. coli*, DNA goes into a co-crystallization state with the stress-induced protein Dps offering protection to several types of stress, which are ordinarily chemical damage^29^. However, while crystallization is often associated with less flexibility or added fragility with direct physical contact, the less compacted DNA of proliferating *E. coli* is elastic and soft^30^, which may limit the number of DSBs. It is then possible that mineral fibres under friction can break DNA strands more easily in the stationary than in the exponential phase.

### Sepiolite fibres can penetrate bacteria when friction forces are present

**Figure 5:**
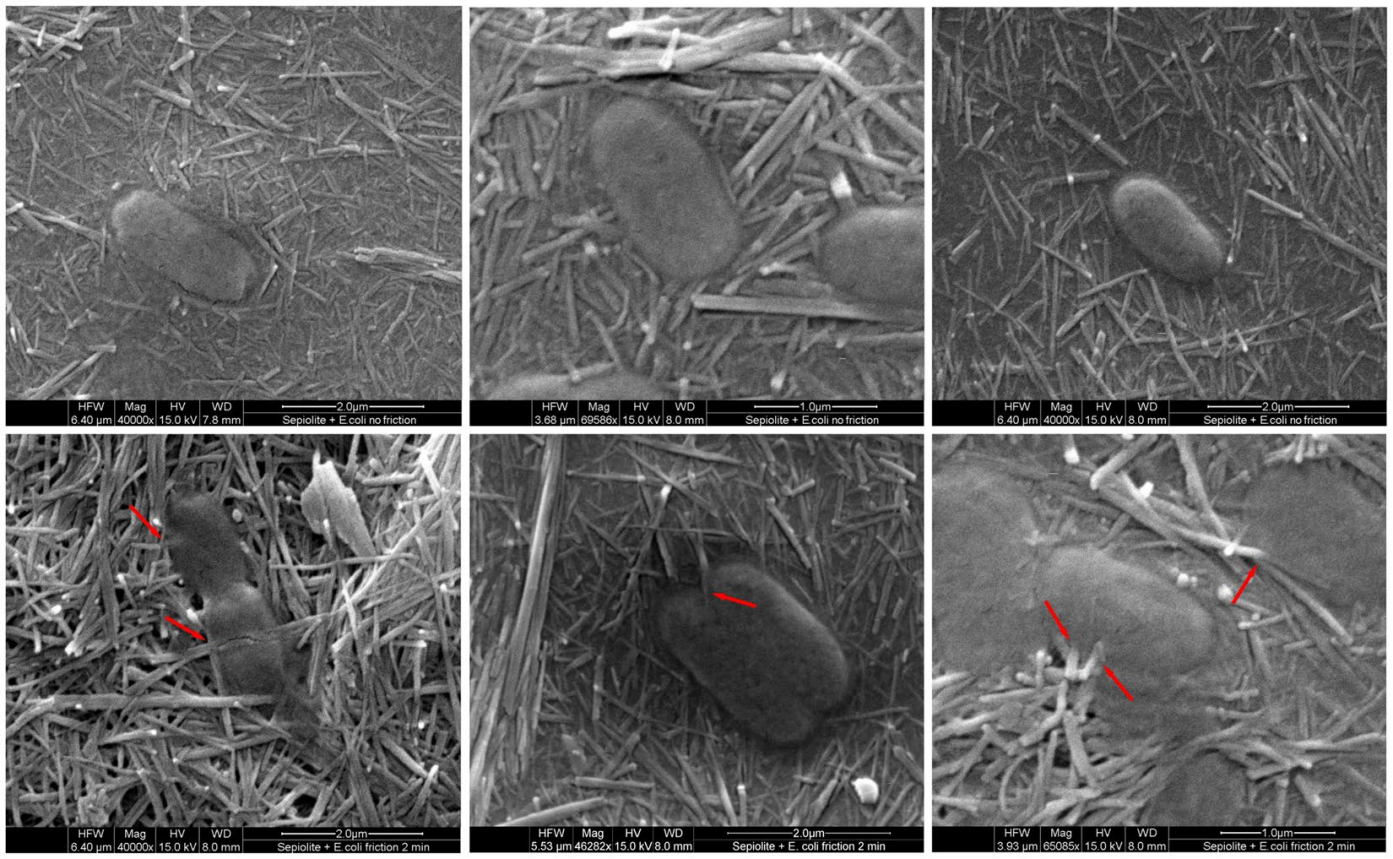
Sepiolite can penetrate bacterial cells when friction forces are applied. SEM of stationary phase *E. coli* MG1655 treated with sepiolite. Red arrows represent potential sites of sepiolite fibre penetration. Bacteria were observed with different magnifications ranging from 40 000X to 70 000X.

To support the hypothesis that the penetration and interaction of fibres with DNA cause DSBs inside the cell, we performed direct observation of sepiolite-treated bacteria using scanning electron microscopy (SEM). Observations from the SEM are compatible with the explanation that fibres are able to penetrate the bacteria without completely destroying the envelope. Additionally, bacteria were directly penetrated by fibres while those that were exposed to mineral without friction were not (Fig. 5). This observation is in concordance with previous studies, whereas sepiolite and other nano-sized acicular materials can penetrate bacterial cells under friction forces on a hydrogel^6^. The partial destruction of the cell wall and the presence of mutants after adding 2-2′ bipyridyl, point to mechanical action as the causative agent of the damage. The notion of mechanical breaks is in agreement with the results from cell-free systems, where the breakage of plasmid DNA was not directly associated with the amount of iron released by asbestos fibres when they were incubated together^14^.

### Sepiolite fibre length matters to cause significant DNA damage in the cell

**Figure 6:**
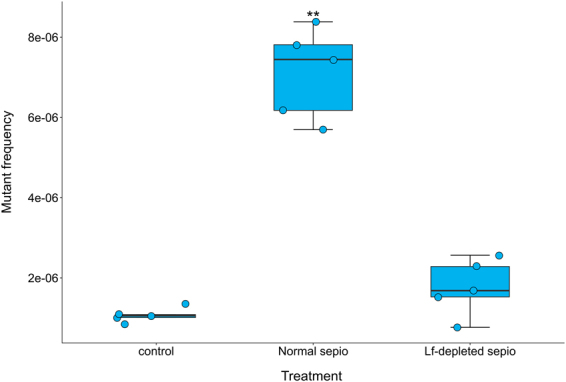
Removal of sepiolite fibres longer than 1 μm decreases fibre-induced mutagenesis to the level of the control. Box-plot of the mutant frequency of *E. coli* MG1655 when sepiolite fibres longer than 1 µm were removed in the mutagenesis experiments (lf-depleted sepiolite). Dry and reconstituted sepiolite (normal sepiolite) and bacterial cells (labeled as control) with no sepiolite were used to compare the effects of long fibre removal. Asterisks represent significant difference; Mann-Whitney *U*: P < 0.01.

Sepiolite also contains both long and short fibres (Fig. S3). In the case of asbestos, long fibres are more dangerous because they have a higher carcinogenic potential. In an experiment designed to test the influence of sepiolite fibre length on mutagenesis in bacteria, we found that the exposure of stationary phase bacteria to a suspension of short fibres (less than 1 µm) did not cause any significant DNA damage when compared with the control, which is in contrast to the long-fibre original mineral suspension (Kruskal-Wallis test; P = 0.005; Fig. 6).

### Asbestos fibres increase the mutant frequency in the same way as sepiolite does

**Figure 7:**
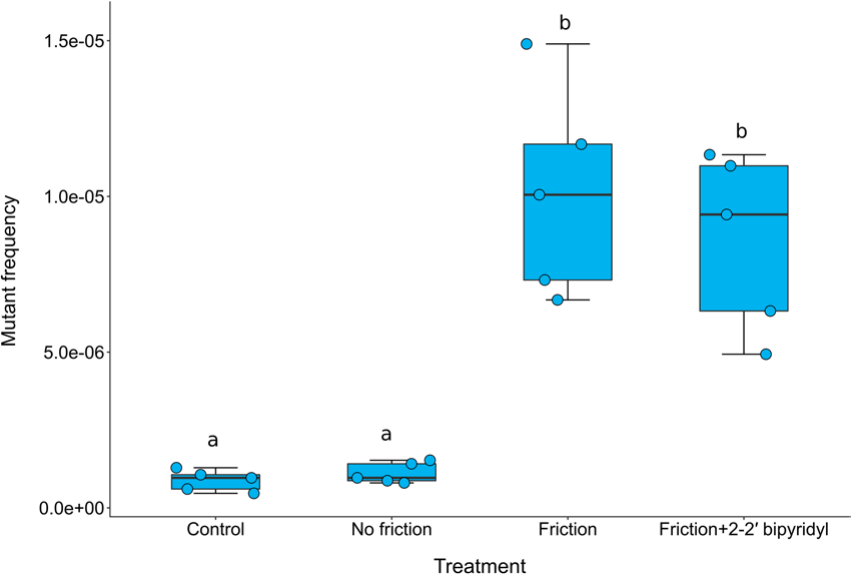
Asbestos can increase the mutation frequency of *E. coli* after friction by around one order of magnitude. Box-plot of mutant frequency induced by asbestos (crocidolite fibres) treatment in *E. coli* MG1655. The asterisk represents a significant difference; Mann-Whitney *U*: P < 0.05. Equal letters represent no differences while different ones represent significant differences.

**Figure 8:**
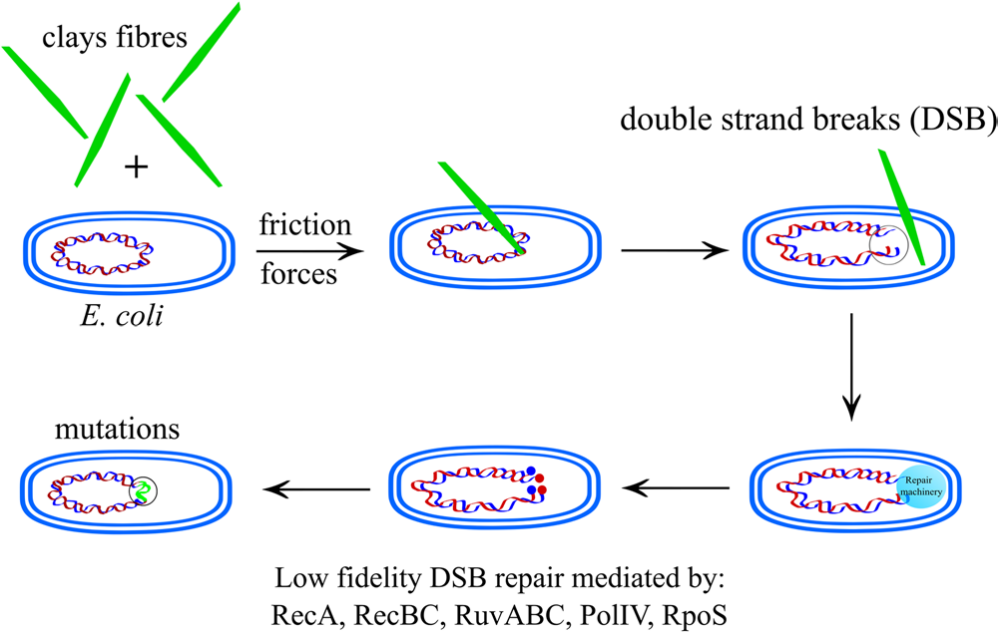
Graphical cartoon model on how clays fibres could induce mutagenesis in bacteria if they are present along with friction forces. The model focuses on *Escherichia coli*.

Bacterial genotoxicity experiments are considered a key step in the assessment of the mutagenic properties of chemicals, drugs or materials in general^31^. Because asbestos fibres resemble sepiolite ones, an experiment to test if asbestos fibres provoke an increase in mutagenesis was designed using crocidolite asbestos (Fig. S3). In our assay, the addition of asbestos to bacteria on plates without friction did not increase the mutant frequency. In contrast, the application of friction when the fibres were present increased the mutant frequency even more than sepiolite alone (Kruskal-Wallis test; P = 0.002; Fig. 7), probably by the same mode of action. Yoshida *et al*. have suggested that asbestos and other clays can be potentially mutagenic based on the integrity analysis of genomic DNA from treated bacteria^32^. We sumarise these results in a simple graphical model for *E. coli* that could be extended to other bacterial types (Fig. 8). A clear antecedent of the ability of fibrous nanoclays to penetrate bacteria was the transformation of monkey cells in culture by exogenous plasmid DNA using chrysotile (a type of asbestos)^10^. Although the procedures are not described in details, we hypothesise that this transformation requires penetration of the cell membrane. Finally, we conducted an additional experiment to discard that friction itself was not responsible for the DNA damage that we observed during this work.

### Further discussion

The poor correlation between DNA damage *in vivo* and *in vitro* described in previous studies^12^ may be explained by the limited or lack of penetration of asbestos in experimental designs. Thus, the introduction of some friction or shaking could be useful in determining if penetration of cells by asbestos and other fibres underlie a molecular mechanism of carcinogenesis. The mechanism(s) underlying asbestos toxicity associated with the pathogenesis of mesothelioma has been a challenge to unravel for more than six decades^33^. According to our results and the current knowledge about asbestos-induced carcinomas, we speculate on a model that explains a potential path leading to carcinomas. Briefly, we think that people exposed to asbestos fibres for prolonged periods accumulate them in the respiratory tract. Asbestos fibres are frequently found in the pleural cavity, and maybe they increase the friction coefficient in the pleural space, a parameter with a very small value in in physiological conditions^34^. The coelomic movement (a cyclical mechanical movement between the parietal pleura, which is the membrane covering the inner surface of the thoracic cavity, and the visceral pleura, which is the membrane covering the lung surface) provokes the movement of asbestos, occasionally trespassing through the mesothelial cell membranes or floating mesothelial cells, physically interacting and disrrupting the DNA or spindle. This physical interaction, with adequate intensity, could induce DSBs, which generate chromosome aberrations or fragmentations in eukaryotic cells as we found here for bacteria. After years of exposure, DSBs or spindle disruption can cause chromosome damages or losses or aneuploidy that increase the probability of malignancy. The proposed model for eukaryote cells would need *in vitro* validation with epithelial cells but this is beyond the scope of the current study. Moreover, this model does not exclude other toxic and genotoxic mechanisms of asbestosis such as reactive species arising from the metal action or an inflammatory response.

One of the most important limitations of our study is the lack of an animal model to test if our finding of mutagenicity in bacteria by clays occurs *in vivo*. In theory, clays present in livestock feed could promote antibiotic resistance and virulence in pathogenic bacteria by not only their transformation ability but also via mutations. However, testing conditions are hindered by the fact that experiments would require at least S1 security level, and this is difficult to achieve with livestock animals^4^. Transformation of plasmid DNA requires penetration, and sepiolite and other clays have shown this capacity in a wide range of concentrations, although it diminishes at high concentrations due to bacterial death^9,35–37^. In a previous study, the pressure in the gut of many animal species was discussed, and these pressures meet the criteria necessary for penetration of bacteria by clay fibres very well^4^. The presence of a hydrogel does not seem to be a problem since both the mucin gut layer or mucoid secretion in the respiratory tract can play that role, particularly if the fibres have the capacity to change local viscosity or if viscosity gradients exist across these body compartments.

An implication of our study is that factors such the friction forces should be considered in assessing the genotoxic and carcinogenic potential of certain fibrous materials. Until now, many studies associate clay-induced damage mostly with ROS^14^. DNA damage can be produced by oxidation-reduction processes generated by metal containing-fibres. Asbestos fibres are carcinogenic for both humans and experimental animals because asbestos produces DNA breaks leading to the formation of a micronucleus (a type of chromosomal aberration)^38^. This kind of damage seems to be caused more by mechanical action rather than ROS generation, which can worsen the situation but is not necessarily the determining factor. In other words, we think that ROS is more a symptom than a cause. Other examples of a potentially dangerous material are the carbon nanotubes (CNTs), a novel industrial material with many applications. The genetic alterations provoked by these nanotubes in rat malignant mesothelioma were similar to those induced by asbestos^39^. Interestingly, CNTs lack heavy metals in their composition. The nanoscale size and needle-like rigid structure of CNTs appear to be associated with their pathogenicity in mammalian cells^39^. Coincidentally, CNTs can be used to transform bacteria with plasmids^40^ in a similar fashion that asbestos^10,41^ and sepiolite do^6,35^. It would not be surprising if these fibrous nanomaterials share their ability to mechanically induce DSBs.

Recently, a possible link between talcum powder and ovarian cancer risk associated with asbestos contamination in talc has been under discussion. Although the risk is small, some studies suggested a low or moderate but significant chance of cancer, while other rejected/discarded this correlation^42–44^. It is necessary to advance the understanding of the molecular bases of DNA damage by asbestos and other industrial fibres. If the proposed model of mechanical/physical DNA breaks is validated in future studies, some genotoxicity assays intended to unveil the mutagenic properties of materials (e.g. the Ames test) should be modified accordingly to include a standardised friction procedure or shaking during incubation steps. Similarly, several *in vitro* tests, with both bacteria and eukaryotic cells, were modified by researchers and regulatory agencies that introduced the metabolic activation by fraction S9 of liver homogenate^45^.

The induction of DSBs by nanofibres in bacteria could also be related to the microbiota evolution of animals that use gastroliths. It has been suggested that gut microbes play a crucial role in keeping species apart or enhancing speciation^46^. It is tempting to speculate that animals that use gastroliths or sediment ingestion expose their microbiota to the abrasive action of stone derivative fibres. Therefore, the shaping of their own microbes is expected to contribute to their own speciation trajectories. Among animals that use or used gastroliths in their evolutionary trajectories, we find several branches of fishes, amphibians, reptiles (including dinosaurs) and birds. Gastroliths also regularly occur in several groups of invertebrates^47^. Wings (2007) recommends making a distinction between lithophagy and geophagy. Lithophagy (stones larger than 0.063 mm in diameter) is defined as the deliberate consumption of stones that turn into gastroliths after their ingestion. Geophagy is the consumption of soil and is known for reptiles, birds, and mammals. These soils, rich in clays, salts or fat, serve mainly as a food supplement for the supply of specific minerals or for medical purposes^47^. Both concepts can contribute the components that this mechanism needs to operate: gut microbiota, gut mucin mucoid layer (hydrogel) and friction forces provided by the peristaltic pressure of the digestive tract in animals, especially the gizzard and the stomach. An interesting question is why sepiolite from limestone gastroliths does not damage the animal gut. A convincing explanation is that the mucoid layer in the gut protects it from the action of these sharp fibres at the time that serves as a protective layer for the gut epithelium. In mammals, this mucoid layer is around 200 µm thick and is under continuous renovation^48^. Sepiolite is a natural clay mineral characterised by a nanofibre structure with average dimensions less than or equal to 0.2 micrometers in diameter, and from 2 to 5 micrometers in length, although longer fibres can be present.

### Concluding remarks

Overall, one of the most significant contributions of this article is the proposition for the first time of a bacterial model to test genotoxicity of nanofibres and to uncover a new mechanism of action for asbestos that correlates better with *in vivo* observations. Asbestosis is a global health and environmental problem, the molecular basis of which has provided a challenge for several decades^33^. Although asbestos fibres are widely distributed in the anatomy of patients^33,49^, the most common cancers caused by asbestos originate in lungs (mostly mesothelioma). If the most explored mechanism of action is based on reactive radicals (chemical damage), why do exposed populations not also show an increased frequency of other types of carcinoma such as leukaemia, lymphoma, liver or kidney cancer? Last but not least, is the tighter contact of slippery membranes (a monolayer of flattened epithelial-like cells) of the mesothelium. The pleural space is in continuous movement and constitutes a preferential target for asbestos-induced carcinogenesis. Of particular interest are free-floating mesothelial cells of the cavity, that even proliferate under damaging conditions^50^. The free-floating cells are the ideal candidates to be penetrated by asbestos in the pleural space. They may be more sensitive to suffering direct (physical) or indirect (chemical) DNA damage and develop into a mesothelioma. Finally, the sepiolite transformation technique gained in popularity in recent years because there is no need to prepare competent cells^9,19,35,51^. In this case, diverse bacteria can be transformed^4^ in both stationary and exponentially growing phases. However, to prevent undesired mutations in both, plasmid and genomic DNA, it is highly recommendable to use exponential phase bacteria, where mutagenesis is not significantly increased, at least in *E. coli*.

## Methods

### Bacteria and growth conditions

The *E. coli* MG1655 wild-type strain and its derivative mutants were cultured in Lysogenic Broth (LB). All experiments were performed at 37 °C, with shaking in liquid culture. All solid cultures were grown in LB agar 1.5% for standard procedures and 2% for the sepiolite treatment. All cultures were supplemented with antibiotics when appropriate.

### Mutant frequency estimation of sepiolite treated cells

Approximately 2 × 10^9^ bacterial cells per ml of *E. coli* MG1655 and its derivative mutants from overnight or mid-exponential growing cultures were centrifuged and resuspended in 100 μl of sterilised transformation mixture, consisting of spanish sepiolite (Kremer Pigmente, Germany) suspended in aqueous solution at a final concentration of 0.1 mg/ml. In addition, to observe the influence of sepiolite concentration in mutant frequency, other concentrations were checked only with overnight cultures (0.1, 1, 10 and 100 mg/ml). Resuspended cells were spread on plates containing fresh Müller-Hinton-Agar (Sigma-Aldrich, Germany) medium solidified with 2% agar, and Petri dishes were pre-dried in a biological safety flow cabinet for 20 minutes before use. Friction force was provided by streaking bacterial cultures plus sepiolite with a sterile glass stirring sticks gently pressed onto the medium surface for one, two and three minutes, applying as much pressure as possible without breaking the agar gel. An additional control experiment was carried out without sepiolite to discard that only friction could induce any DNA damage in this conditions. Petri dishes were incubated at 37 °C for 2 hours to allow for DNA repair if any damage had occurred. The plates were gently washed four times with 5 ml of 0.9% sodium chloride solution using a 5 ml pipette and the resulting bacterial suspensions were transferred to 10 ml tubes to recover the cells by centrifugation at 3000 g for 10 minutes. The resulting pellets were resuspended in a final volume of 1 ml of fresh LB an incubate for 1 hour at 37 °C to allow the cells to recover. Appropriate dilutions were plated onto LB plates to estimate bacterial viability and in LB plus the antibiotic fosfomycin (50 µg/ml) to estimate the number of resistant mutants. Plates were incubated overnight at 37 °C. Each experiment consisted of 5 replicates and was repeated twice. Mutant frequencies were calculated by using the FALCOR web-tool^52^.

### Influence of 2-2′ bipyridyl on sepiolite mutagenesis

The effect of 2-2′ bipyridyl, a metal chelating agent^53^, on sepiolite mutagenesis was determined by measuring its influence on the mutant frequency for a selected concentration of sepiolite, where mutagenesis was observed. The experiment consisted of adding a titrating concentration of 2-2′ bipyridyl (200 µM) to chelate metals five minutes before the treatment. Cultures treated with sepiolite and friction without the addition of 2-2′ bipyridyl and bacteria alone without sepiolite were used as a control. The mutant frequencies for these groups were determined as described above.

### Assessing double-strand breaks with a plasmid system

To evaluate if sepiolite under friction treatment induces double-strand breaks in plasmid DNA, the strain *Escherichia coli* DH5α (fhuA2 lac(del)U169 phoA glnV44 Φ80′ lacZ(del)M15 gyrA96 recA1 relA1 endA1 thi-1 hsdR17) carrying the plasmid pET-19b (Novagen, Germany) was treated with sepiolite and sliding friction forces for one minute. Several samples were recovered from the plates and pooled to compensate for viability losses due to friction. The recovery was done by washing the surface with 5 ml 0.9% NaCl saline solution four times as described for mutagenesis experiments. The recovered pellets were washed with 1 ml of TE buffer and the OD_600_ adjusted to 1 for each type of sample. Plasmid DNA samples were extracted using a Qiagen mini plasmid extraction kit (Qiagen, Germany). Added sepiolite with or without friction and no sepiolite groups were used as a control group. Each experiment consisted of five replicates. The same amount of plasmid DNA per replicate was applied per well to an agarose gel that was stained with SYBR® Gold Nucleic Acid Gel Stain kit (Molecular Probes, USA). A NdeI (Promega, USA) digested aliquot of pET-19b was used as a control for the linear migration rate. The proportion of linear molecules of the plasmid were compared among groups using densitometry analysis with ImageJ^54^.

### RecA deficient strain construction

The *recA* null mutant was constructed following a previously described methodology^55^ with the primers 5′-CAGAACATATTGACTATCCGGTATTACCCG-GCATGACAGGAGTAAAAATGGT-GTAGGCTGGAGCTGCTTC-3′ and 5′-ATGCGACCCTTGTGTATCAAACAAGACGATTAAAAATCTTCGTTAGTTTCATGGGAAT-TAGCCATGGTCC-3′ (forward and reverse respectively) using the pKD3 plasmid as template. The mutant was checked by PCR amplification using the primers c1 5′-TTATACGCAAGGCGACAAGG-3′ and c2 5′-GATCTTCCGTCACAGGTAGG-3′ in combination with specific primers for upstream and downstream regions of *recA* gene: 5′-ATTGCAGACCTTGTGGCAAC-3′ and 5′-CGATCCAACAGGCGAGCATAT-3′ respectively. Additionally, the increased susceptibility to UV light and mitomycin C was tested phenotypically in comparison to the parental strain. The antibiotic resistance gene was eliminated using the pCP20 plasmid as described previously^55^.

### SEM of *E. coli* treated with sepiolite

Approximately 2 × 10^9^ CFU of stationary phase *E. coli* MG1655 were treated with sepiolite and friction force was applied for one minute as described for the mutagenesis experiment. Circular agar blocks were taken from agar plates with a sterile cork borer (1 cm of diameter). Then, a thin surface layer was cut off, placed on a circular glass cover slip (1.5 cm of diameter) and incubated for 45 minutes at room temperature in a laminar flow cabinet to allow air drying of the samples. The cover slips with dehydrated agar sections were mounted on aluminium stubs using double-sided adhesive tape and coated with gold in a sputter coater (SCD-040; Balzers, Union, Liechtenstein). The specimens were examined with an FEI Quanta 200 SEM (FEI Co., Hillsboro, OR) operating at an accelerating voltage of 15 kV under high vacuum mode at different magnifications. At least 5 fields from independent plates were observed to check physical penetration by the mineral. Some samples of sepiolite or asbestos (crocidolites) alone were processed and observed in the same way.

### Long fibre-depleted sepiolite mutagenesis experiment

To assess the role of sepiolite long fibres in mutagenesis, a sepiolite preparation depleted of fibres longer than 1 µm was obtained. A 100 ml sepiolite suspension (1 mg/ml) in distilled water was passed through Pall® Acrodisc® glass fibre syringe filters (Sigma, USA) several times. The resulting suspension was desiccated by evaporation at 70 °C overnight. A non-filtered solution was used as a control. From the obtained powder, two suspensions were prepared to a final proportion of 0.1 mg/ml. These two solutions were used for a mutagenesis experiment plating with fosfomycin as indicated previously, using a friction time of two minutes.

### Mutant frequency estimation of asbestos treated cells

The procedure was carried out identically to the one described above for sepiolite. The friction time was set to two minutes and the same concentration that was used, 0.1 mg/ml of asbestos. We used the crocidolite asbestos analytical standard (SPI Supplies, USA). The asbestos fibres were resuspended in distilled water, autoclaved and sonicated in a water bath for 10 minutes before use to produce a homogeneous suspension.

### Statistical analysis

To compare experimental groups, Kruskal-Wallis tests or One-way ANOVA tests were performed. In the case of significance, Bonferroni-corrected one-tailed Mann-Whitney U test or Tukey HSD test was used respectively. For the analysis of dose-response curves, we used the disease control rate (DCR) package of R sofware. The P values less than or equal to 0.05, after correction if needed, were considered statistically significant. All tests were performed with the statistical software R v. 3.4.2^56^.

## Electronic supplementary material

Supplementary information
