# Supplementary material for: Clay-induced DNA breaks as a path for genetic diversity, antibiotic resistance, and asbestos carcinogenesis
Source: Sci Rep. 2018 May 31;8:8504. doi: 10.1038/s41598-018-26958-5 (PMC5981458; doi:10.1038/s41598-018-26958-5)

Supplementary material

Clay-induced DNA breaks as a path for genetic diversity, antibiotic resistance, and asbestos carcinogenesis

Enrique González-Tortuero[1,2,§], Jerónimo Rodríguez-Beltran[3], Renate Radek[4], Jesús Blázquez[3] and Alexandro Rodríguez-Rojas[4,*]

[1]Department of Ecosystem Research, Leibniz-Institute of Freshwater Ecology and Inland Fisheries (IGB), Müggelseedamm 301, 12587 Berlin, Germany.

[2]Berlin Centre for Genomics in Biodiversity Research (BeGenDiv), Königin-Luise-Straße 6-8, 14195 Berlin, Germany.

[3]Institut für Biologie, Freie Universität Berlin. Königin-Luise-Str. 1-3 14195 Berlin, Germany.

[§]Current Address: Institute for Genome Sciences, University of Maryland Baltimore School of Medicine, 670 West Baltimore Street, 21201 Baltimore, MD, USA.

[4]Evolutionary Biology, Institut für Biologie, Freie Universität Berlin, Berlin, Germany

[*]Author to whom correspondence should be addressed: a.rojas@fu-berlin.de

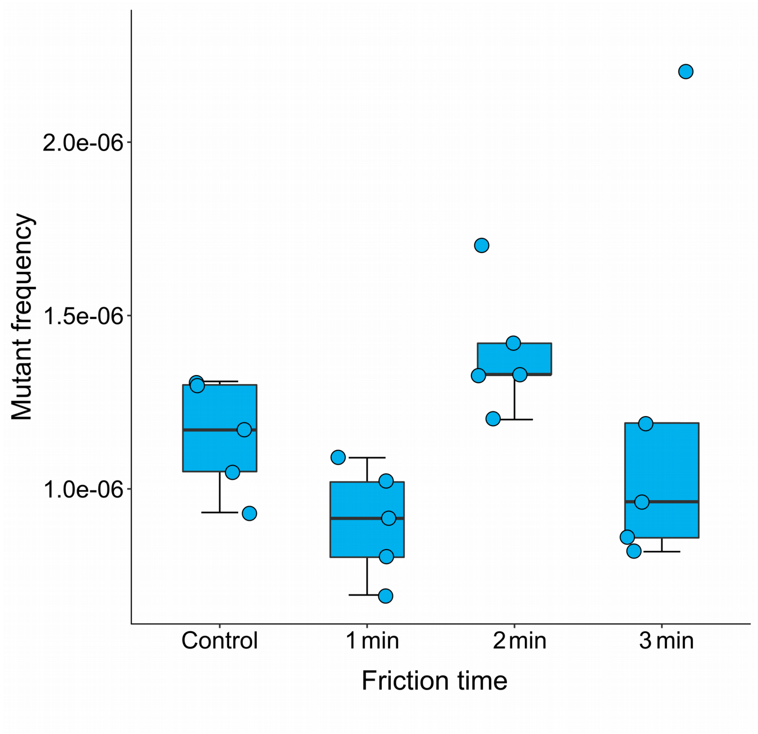

Fig S1. The action of applying friction forces alone (without the addition of clays) does not significantly contribute to mutagenesis. Box-plot of the mutant frequencies of *E. coli* MG1655 after different times of friction. No significant differences were found; Mann-Whitney U: P > 0.05.

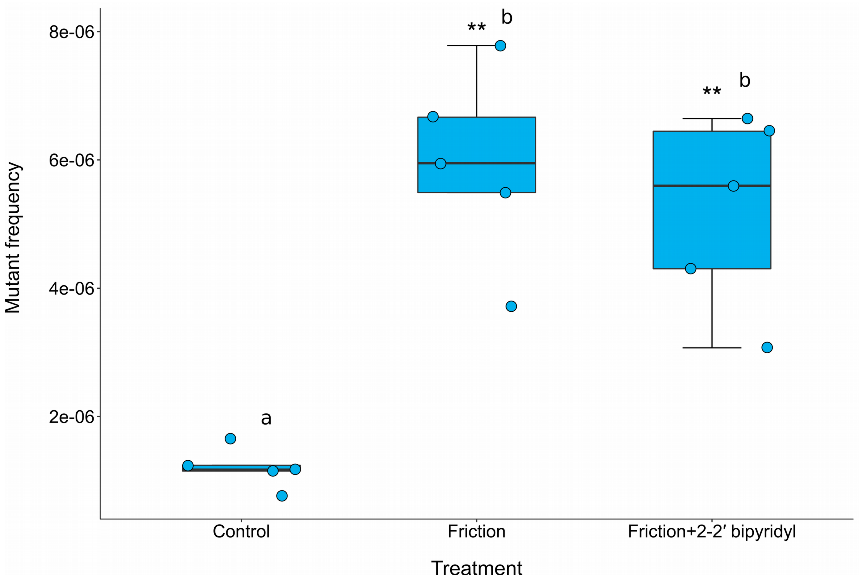

Fig S2. Addition of a chelating agent (2-2' bipyridyl) does not significantly suppress or diminish the mutagenic effect of sepiolite. Box-plot of mutant frequency of *E. coli* MG1655 when added 2-2' bipyridyl as chelating agent. The asterisks represent significant differences; Mann-Whitney U: P < 0.01. Equal letters represent no differences while different ones represent significant differences.

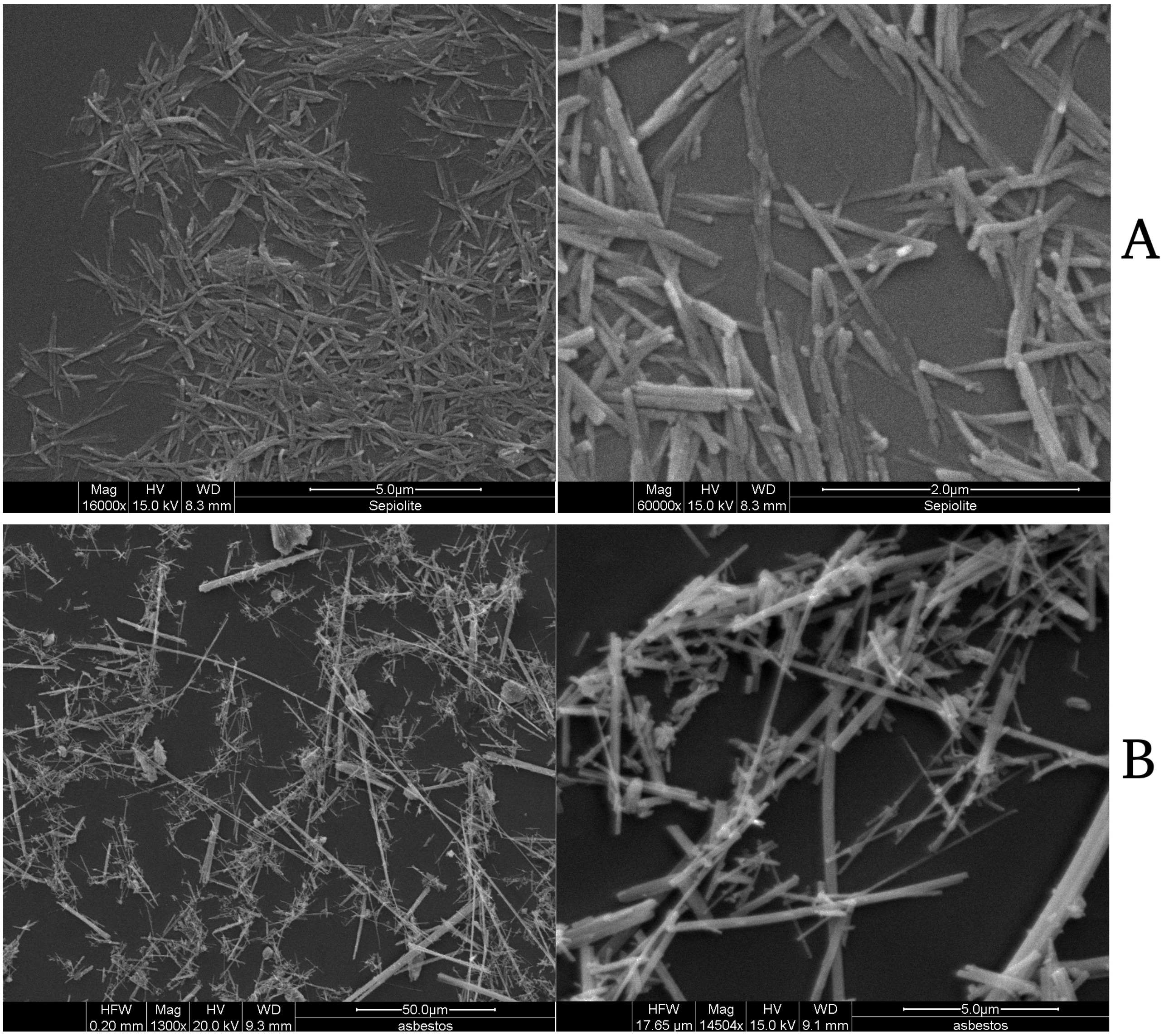

Fig S3. Visualisation of sepiolite and asbestos fibres under SEM. SEM examination of (A) sepiolite fibres and (B) asbestos. Fibres were observed at different magnifications as indicated at the bottom of the pictures.

Fig S4. Full picture of the gel presented in the figure 3. Different exposure times and modes (direct or inverted gray scale colours)

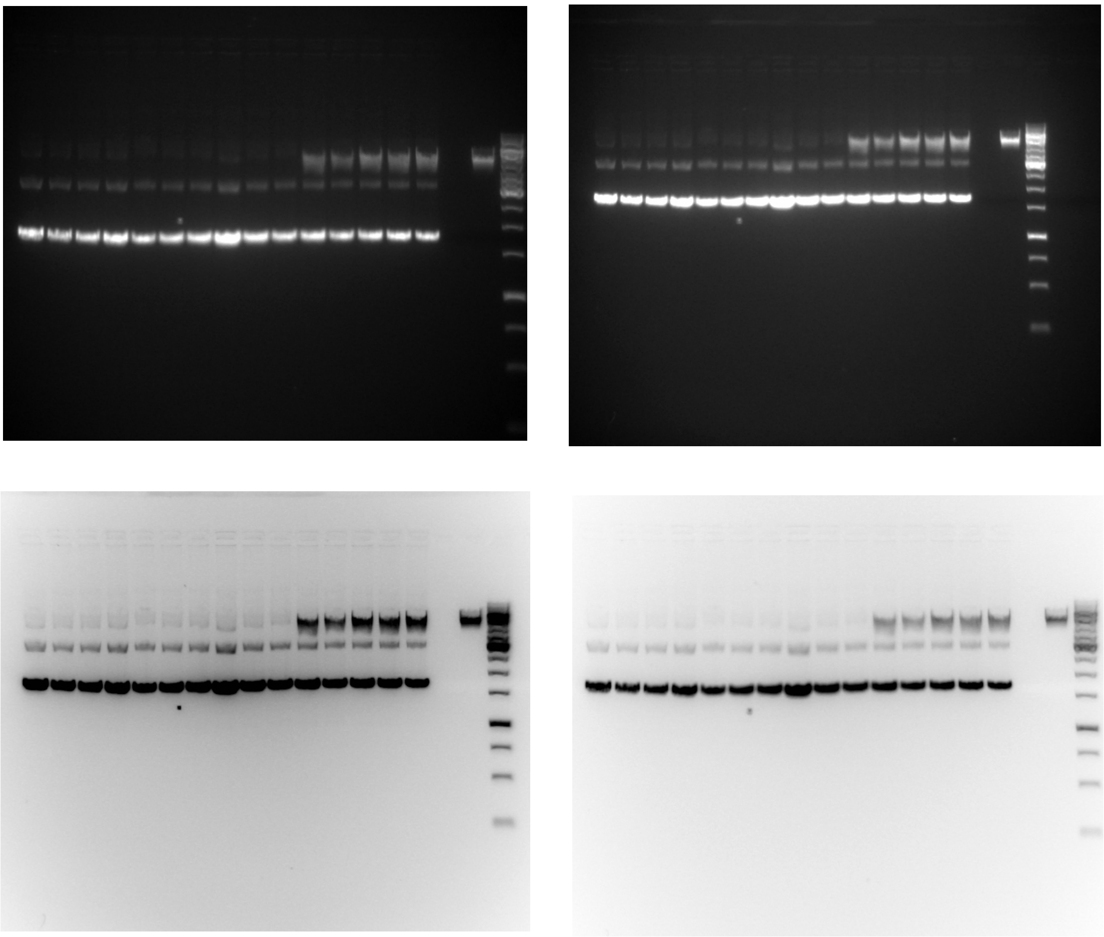

Supplement: Supplementary file 1 — Supplementary information [file 41598_2018_26958_MOESM1_ESM.pdf]
